# Clinical analysis of Meckel diverticulum with ectopic pancreas in children: 3 case reports

**DOI:** 10.1097/MD.0000000000047794

**Published:** 2026-02-28

**Authors:** Guanyu Lai, Junmei Ma, Fang Hou, Bing Xu

**Affiliations:** a Department of Pediatric Surgery, Sichuan Provincial People's Hospital, School of Medicine, University of Electronic Science and Technology of China, Chengdu, China; b Department of Pediatric Surgery, Sichuan Provincial People's Hospital, Chengdu, China.

**Keywords:** case report, child, ectopic pancreas, laparoscope, Meckel diverticulum

## Abstract

**Rationale:**

Lower gastrointestinal bleeding caused by Meckel diverticulum (MD) with ectopic pancreas is rare and diagnostically challenging in children, particularly due to the frequent absence of gastric mucosa leading to negative Technetium pertechnetate. This study aimed to summarize and analyze the clinical characteristics and management of this condition.

**Patient Concerns:**

A retrospective analysis included 3 pediatric patients (2 males, 1 female; aged 2–13 years) treated between July 2013 and March 2023. All presented with hematochezia as the primary symptom. One case was accompanied by abdominal pain, and another presented with sudden syncope.

**Diagnoses:**

Technetium pertechnetate was negative in all three patients. The definitive diagnosis of MD was established intraoperatively via diagnostic laparoscopy in all cases. Postoperative histopathological examination confirmed the presence of ectopic pancreatic tissue within the resected diverticula.

**Interventions:**

All patients underwent diagnostic laparoscopic exploration, which confirmed the MD. A laparoscopic-assisted diverticulectomy was subsequently performed.

**Outcomes:**

The surgical intervention completely resolved hematochezia in all children. Postoperative recovery was uneventful, with patients resuming oral intake on postoperative day 3 and being discharged 4 to 5 days after surgery. Follow-up at 1, 3, and 6 months showed no recurrence of symptoms, and abdominal ultrasounds revealed no abnormalities.

**Lessons:**

MD with ectopic pancreas in children lacks specific clinical manifestations. Preoperative diagnosis is challenging, as the absence of gastric mucosa often results in negative Technetium pertechnetate. Laparoscopic exploration serves as a valuable minimally invasive approach that provides both definitive diagnosis and therapeutic management in cases with high clinical suspicion.

## 1. Introduction

Meckel diverticulum (MD) is the most common congenital malformation of the gastrointestinal tract, resulting from the incomplete obliteration of the omphalomesenteric (vitelline) duct during embryonic development.^[1]^ It is a true diverticulum, containing all layers of the intestinal wall. While ectopic tissue is found in 50% to 60% of symptomatic cases, typically gastric mucosa, the presence of ectopic pancreatic tissue as the predominant or sole heterotopic tissue is relatively uncommon.^[2,3]^ This report analyzes the clinical presentation, diagnostic challenges, and management of 3 pediatric cases of MD in which the primary ectopic tissue was pancreas.

We retrospectively reviewed the medical records of 3 children diagnosed with MD containing ectopic pancreatic tissue who were treated in our department. Data collection included clinical presentation, physical findings, laboratory and imaging results, surgical procedures, pathological findings, and postoperative outcomes.

This retrospective study was approved by the Ethics Committee of Sichuan Provincial People’s Hospital (Approval No.: 2024-209). The title is diagnosis and treatment analysis of MD with simple ectopic pancreas in Children. The need for informed consent was waived by the Sichuan Provincial People’s Hospital Ethics Committee due to the retrospective nature of the study. And Sichuan Provincial People’s Hospital has authorized this study to use relevant data.

## 2. Clinical presentation

We enrolled patients admitted to the Department of Pediatric Surgery at Sichuan Provincial People’s Hospital between 2013 and 2023, in whom MD was identified intraoperatively and histopathological examination revealed the presence of pancreatic tissue without other abnormalities. None of the children had a positive family history for related symptoms or disorders. The detailed clinical characteristics of all patients can be found in Table 1.

**Table 1:** Clinical characteristics of the 3 pediatric patients with Meckel diverticulum and ectopic pancreas.

characteristics	Patient 1 (female, 2yr)	Patient 2 (male, 4 yr)	Patient 3 (male, 13 yr)
Main symptom	Hematochezia/2 d, dark red/3–4 d, bright red/1 d	Hematochezia/4 d, bright red/4–5 d	Hematochezia/10 d, dark red/4 d
Other symptoms	Abdominal pain, restlessness	Dizziness, fatigue	Sudden syncope (1 episode)
Preop bleeding vol	500 mL/d	500–700 mL/d	400–600 mL/d
Vitals/signs	BP 95/64 mm Hg, marked periumbilical tenderness	BP 89/58 mm Hg, mild periumbilical tenderness	BP 100/65 mm Hg, mild periumbilical tenderness
Lab tests	RBC 3.62 × 10^12^/L, HGB 71 g/L	RBC 1.34 × 10^12^/L, HGB 41 g/L	RBC 2.64 × 10^12^/L, HGB 68 g/L
Tc^99^m scan	Negative	Negative	Negative
Preop transfusion	800 mL	1500 mL	700 mL
Diverticulum size	3 × 2.3 cm	6 × 4 cm	10 × 3 cm
Pathology findings	Meckel diverticulum with pancreatic tissue	Meckel diverticulum with abundant pancreatic tissue in submucosa	Meckel diverticulum with pancreatic tissue

The clinical details of the 3 patients are summarized in Table 1. All patients were admitted due to hematochezia. The duration of symptoms ranged from 2 to 10 days. One patient had predominantly bright red blood per rectum, while 2 had dark red stools. The frequency was 3 to 5 times per day, with an estimated volume of 100 to 150 mL per episode. One patient experienced persistent and progressively worsening periumbilical dull pain. Another patient’s initial symptom was sudden syncope. No patients reported hematemesis, abdominal distension, diarrhea, or fever. There was no significant previous surgical history.

## 3. Physical examination

All patients exhibited varying degrees of hypotension and tachycardia, accompanied by pallor. No skin petechiae, mucosal hemorrhages, or ecchymoses were observed. Cardiopulmonary examination was unremarkable. Abdominal examination revealed a soft and flat abdomen without visible peristalsis or masses. Tenderness was noted around the umbilicus in all cases, without rebound tenderness. The liver and spleen were not palpable, liver dullness was normal, and no shifting dullness was detected. Bowel sounds were hyperactive. Digital rectal examination showed no obvious abnormalities.

## 4. Laboratory and Imaging findings

Laboratory tests revealed hemoglobin levels between 40 and 70 g/L. Platelet count, white blood cell count, coagulation profile, liver and kidney function tests, and urinalysis were within normal limits. Stool routine examination was positive for occult blood (++++), and no ova were seen. Abdominal color Doppler ultrasound and plain radiographs showed no significant abnormalities. Technetium pertechnetate scintigraphy was negative for all 3 patients. Colonoscopy performed in 1 patient revealed intraluminal blood and active bleeding from the small intestine at the ileocecal valve.

## 5. Treatment and surgical findings

**Figure 1.**
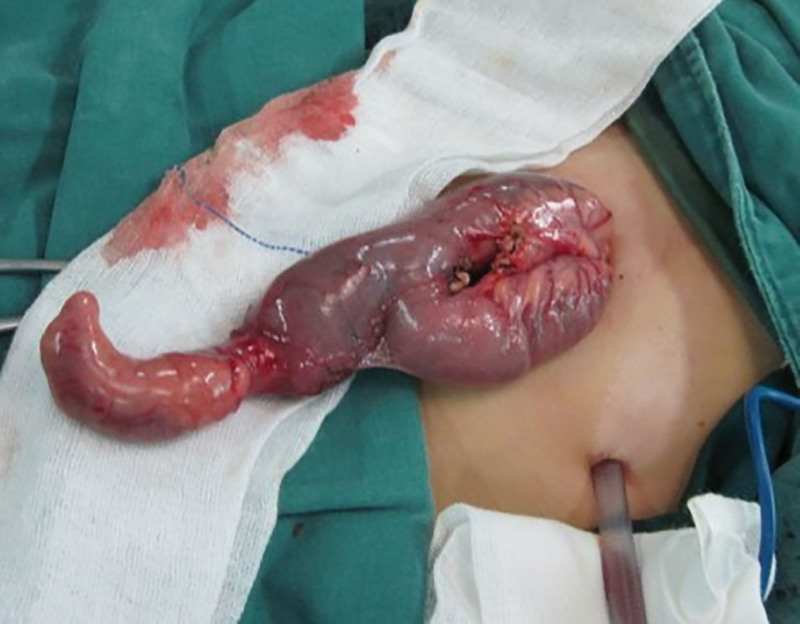
A yellowish-white, thickened and abnormal serosal surface was visible at the apex of the diverticulum.

All patients received initial conservative management, including anti-shock therapy, hemostatic agents, and blood transfusions (patients received multiple preoperative blood transfusions, with total volumes of: 700–1500 mL). Upon admission, all patients presented with pre-shock manifestations, including tachycardia and low blood pressure. After admission, they were immediately placed on nil per os status. The pediatric surgery emergency physician calculated the required total fluid volume based on body weight and initiated fluid resuscitation. Meanwhile, urgent laboratory tests revealed low hemoglobin levels, prompting an emergency blood transfusion. Given the presence of hematochezia, intramuscular Haemocoagulase Agkistrodon for Injection was administered to achieve hemostasis. However, hematochezia persisted, and hemoglobin levels continued to decline progressively. After multidisciplinary discussion and parental consent, diagnostic laparoscopy was performed. Intraoperatively, MD was identified in all cases. The diverticula were located on the antimesenteric border of the ileum, 40 to 60 cm proximal to the ileocecal valve, measuring 3 to 10 cm in length and 2 to 4 cm in width. Whitish pancreatic-like tissue was visible at the apex of the diverticulum (Fig. 1). The distal bowel contained a significant amount of old blood. One patient had a 1 × 1 cm ulcer within the diverticulum. Laparoscopic-assisted diverticulectomy with segmental bowel resection and anastomosis was performed by exteriorizing the affected bowel segment through an extended umbilical incision. Minimal peritoneal effusion was noted in every case during the procedure.

## 6. Pathological findings

**Figure 2.**
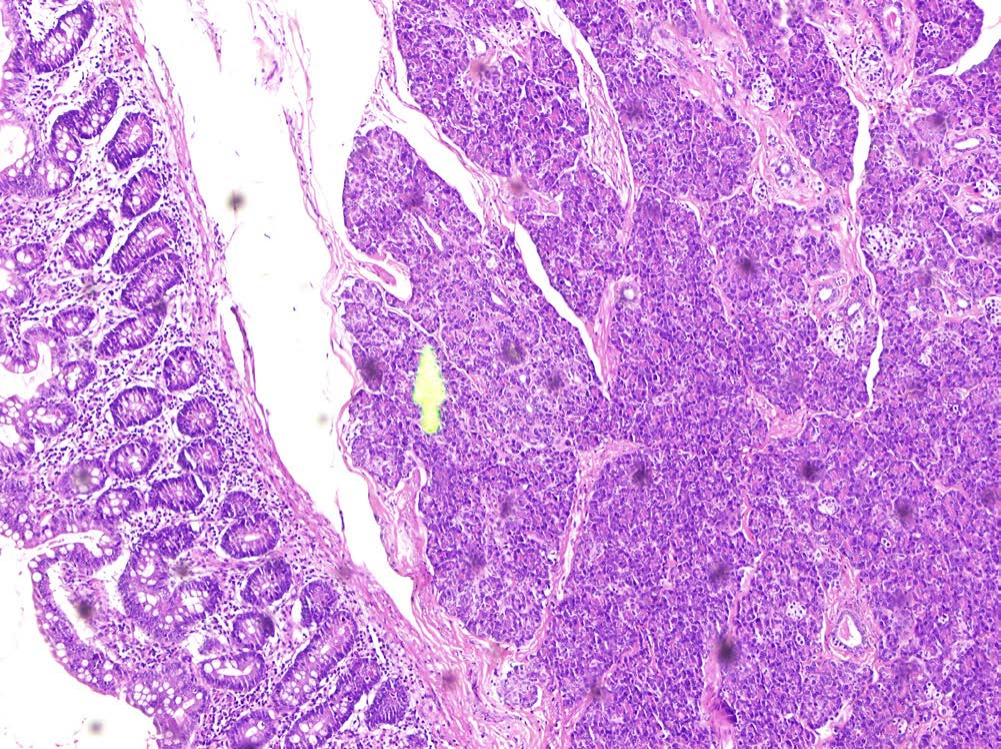
We were able to identify enterocytes and pancreatic cells.

Histopathological examination confirmed the diagnosis of MD in all specimens. Ectopic pancreatic tissue was identified within each diverticulum, primarily located at the apex (Fig. 2). In 1 case, the pancreatic tissue extended from the apex towards the base. The mucosa overlying the ectopic tissue showed areas of erosion. Microscopically, pancreatic acinar tissue with surrounding lymphocytic infiltration was observed, and exocrine ducts were identified in 2 cases.

## 7. Outcomes and follow-up

The mean operative time was 75 minutes. Postoperative recovery was smooth for all patients. Hematochezia resolved completely. Patients started oral intake on postoperative day 3 and were discharged 4 to 5 days after surgery. Follow-up at 1, 3, and 6 months postoperatively revealed no recurrence of hematochezia, abdominal pain. Repeat blood tests and abdominal ultrasounds were normal.

## 8. Discussion

MD occurs in approximately 2% of the population.^[1]^ Symptoms develop in a minority, commonly due to complications associated with ectopic tissue. While gastric mucosa is the most frequent heterotopia (leading to positive Meckel scans), ectopic pancreas is rare.^[2–5]^ The pathogenesis of ectopic pancreas remains unclear but may involve abnormal migration and fusion of pancreatic buds or metaplasia of endodermal tissue during embryogenesis.^[6,7]^ It can occur throughout the GI tract, with MD being an uncommon site (5.3%).^[7]^

The most common clinical presentation of MD in children is lower gastrointestinal bleeding (30%–56%), often painless and recurrent.^[8]^ Our cases presented with significant, acute hematochezia. Ectopic pancreas itself is often asymptomatic, but the secretion of pancreatic enzymes may contribute to mucosal ulceration, inflammation, and bleeding by eroding adjacent blood vessels.^[7,9]^ This mechanism might explain the substantial bleeding observed in our patients. Periumbilical tenderness, noted in all our cases, is an uncommon finding in MD and adds to the diagnostic challenge, possibly related to intraluminal blood distending and irritating the bowel.^[2]^

Imaging diagnosis of MD can be challenging. Ultrasound, while highly sensitive and specific in some studies,^[10]^ was negative in our patients, highlighting its limitations, particularly operator dependency and bowel distension. Technetium pertechnetate scintigraphy is considered the best noninvasive test for MD, but its accuracy relies on the presence of ectopic gastric mucosa.^[11]^ False-negative results, as seen in all our cases, occur when ectopic gastric mucosa is absent or scant, or in the presence of inflammation, necrosis, or massive bleeding.^[11,12]^ Our patients’ negative scans are directly attributable to the predominance of pancreatic tissue and the absence of gastric mucosa.^[13]^ Colonoscopy is typically nondiagnostic but can help exclude colonic sources of bleeding, as demonstrated in 1 of our cases where bleeding from the ileocecal valve was visualized. Small bowel capsule endoscopy shows promise, with a reported diagnostic yield of 82.8% for bleeding MD.^[14]^ Certainly, CT scans can be considered for patients presenting with acute abdomen. Some studies indicate that while CT examinations may yield positive findings in cases of MD, the detection rate does not exceed 50%. However, when contrast-enhanced CT or CT with oral contrast agents is employed, the diagnostic sensitivity for MD can be substantially improved. Therefore, this study suggests that CT examination could be incorporated into the diagnostic workup for acute abdomen or suspected MD.^[15–17]^

The primary differential diagnoses for MD presenting with hemorrhage of lower digestive tract include infectious enterocolitis, intestinal polyps, intestinal duplication, intussusception, and vascular malformations.^[4]^ Distinguishing surgical from medical causes of bleeding is crucial. In our cases, the acute nature of massive bleeding, associated abdominal tenderness, failure of conservative management, and continued hemoglobin decline despite transfusion strongly suggested a surgical etiology. The clinical suspicion for MD, despite negative imaging, warranted surgical exploration.

Laparoscopy plays a dual role as both a diagnostic and therapeutic modality. It offers the advantages of being minimally invasive, providing direct visualization, and allowing for definitive treatment during the same procedure.^[3,9]^ Preoperative diagnosis of ectopic pancreas is exceedingly difficult, and histopathological confirmation is typically obtained post-resection. Laparoscopy avoids the morbidity of a negative laparotomy and facilitates a targeted resection. In our series, laparoscopic-assisted resection via an umbilical incision was safe, effective, and resulted in short hospital stays. The combination of laparoscopy with other diagnostic modalities can enhance the diagnostic accuracy for complex pediatric gastrointestinal bleeding.

## 9. Conclusion

MD containing ectopic pancreas is a rare cause of lower gastrointestinal bleeding in children. The absence of ectopic gastric mucosa often leads to negative Technetium pertechnetate scintigraphy, making preoperative diagnosis difficult. A high index of suspicion is necessary, especially in cases of significant, acute hematochezia unresponsive to medical management. Laparoscopic exploration is a highly valuable approach, offering the benefits of minimal invasiveness, accurate diagnosis, and simultaneous therapeutic intervention, and should be considered when clinical suspicion remains high despite nondiagnostic conventional imaging.

## Acknowledgments

The authors would like to thank you for your support and assistance during the preparation of this manuscript.

## Author contributions

**Conceptualization:** Guanyu Lai, Junmei Ma, Bing Xu.

**Data curation:** Guanyu Lai, Junmei Ma, Bing Xu.

**Formal analysis:** Guanyu Lai, Junmei Ma, Fang Hou.

**Investigation:** Guanyu Lai.

**Methodology:** Guanyu Lai.

**Resources:** Fang Hou.

**Software:** Guanyu Lai, Fang Hou.

**Supervision:** Fang Hou, Bing Xu.

**Validation:** Guanyu Lai, Fang Hou.

**Visualization:** Guanyu Lai, Fang Hou.

**Writing – original draft:** Guanyu Lai, Junmei Ma.

**Writing – review & editing:** Bing Xu.
